# Differentiating IDH-mutant astrocytomas and 1p19q-codeleted oligodendrogliomas using DSC-PWI: high performance through cerebral blood volume and percentage of signal recovery percentiles

**DOI:** 10.1007/s00330-024-10611-z

**Published:** 2024-01-29

**Authors:** Albert Pons-Escoda, Alonso Garcia-Ruiz, Pablo Naval-Baudin, Ignacio Martinez-Zalacain, Josep Castell, Angels Camins, Noemi Vidal, Jordi Bruna, Monica Cos, Raquel Perez-Lopez, Laura Oleaga, Esther Warnert, Marion Smits, Carles Majos

**Affiliations:** 1 Radiology Department, Feixa Llarga SN, Hospital Universitari de Bellvitge, 08907 Barcelona, Spain; 2 Neuro-oncology Unit, Feixa Llarga SN, Institut d’Investigació Biomèdica de Bellvitge- IDIBELL, 08907 Barcelona, Spain; 3 Facultat de Medicina i Ciències de La Salut, Universitat de Barcelona (UB), Carrer de Casanova 143, 08036 Barcelona, Spain; 4 Diagnostic Imaging and Nuclear Medicine Research Group, Institut d’Investigació Biomèdica de Bellvitge- IDIBELL, Feixa Llarga SN, 08907 Barcelona, Spain; 5 Radiomics Group, Vall d’Hebron Institut d’Oncologia- VHIO, Carrer de Natzaret, 115-117, 08035 Barcelona, Spain; 6 Pathology Department, Feixa Llarga SN, Hospital Universitari de Bellvitge, 08907 Barcelona, Spain; 7 Radiology Department, Hospital Clinic de Barcelona, Villarroel 170, 08036 Barcelona, Spain; 8 Department of Radiology & Nuclear Medicine, Erasmus MC, Molewaterplein 40, 3015 GD Rotterdam, The Netherlands; 9 Erasmus MC Cancer Institute, Erasmus MC, Molewaterplein 40, 3015 GD Rotterdam, The Netherlands; 10 Medical Delta, Delft, The Netherlands

**Keywords:** Brain neoplasms, Astrocytoma, Oligodendroglioma, Perfusion imaging, Magnetic resonance imaging

## Abstract

**Objective:**

Presurgical differentiation between astrocytomas and oligodendrogliomas remains an unresolved challenge in neuro-oncology. This research aims to provide a comprehensive understanding of each tumor’s DSC-PWI signatures, evaluate the discriminative capacity of cerebral blood volume (CBV) and percentage of signal recovery (PSR) percentile values, and explore the synergy of CBV and PSR combination for pre-surgical differentiation.

**Methods:**

Patients diagnosed with grade 2 and 3 IDH-mutant astrocytomas and IDH-mutant 1p19q-codeleted oligodendrogliomas were retrospectively retrieved (2010–2022). 3D segmentations of each tumor were conducted, and voxel-level CBV and PSR were extracted to compute mean, minimum, maximum, and percentile values. Statistical comparisons were performed using the Mann-Whitney *U* test and the area under the receiver operating characteristic curve (AUC-ROC). Lastly, the five most discriminative variables were combined for classification with internal cross-validation.

**Results:**

The study enrolled 52 patients (mean age 45-year-old, 28 men): 28 astrocytomas and 24 oligodendrogliomas. Oligodendrogliomas exhibited higher CBV and lower PSR than astrocytomas across all metrics (e.g., mean CBV = 2.05 and 1.55, PSR = 0.68 and 0.81 respectively). The highest AUC-ROCs and the smallest *p* values originated from CBV and PSR percentiles (e.g., PSRp70 AUC-ROC = 0.84 and *p* value = 0.0005, CBVp75 AUC-ROC = 0.8 and *p* value = 0.0006). The mean, minimum, and maximum values yielded lower results. Combining the best five variables (PSRp65, CBVp70, PSRp60, CBVp75, and PSRp40) achieved a mean AUC-ROC of 0.87 for differentiation.

**Conclusions:**

Oligodendrogliomas exhibit higher CBV and lower PSR than astrocytomas, traits that are emphasized when considering percentiles rather than mean or extreme values. The combination of CBV and PSR percentiles results in promising classification outcomes.

**Clinical relevance statement:**

The combination of histogram-derived percentile values of cerebral blood volume and percentage of signal recovery from DSC-PWI enhances the presurgical differentiation between astrocytomas and oligodendrogliomas, suggesting that incorporating these metrics into clinical practice could be beneficial.

**Key Points:**

*• The unsupervised selection of percentile values for cerebral blood volume and percentage of signal recovery enhances presurgical differentiation of astrocytomas and oligodendrogliomas.*

*• Oligodendrogliomas exhibit higher cerebral blood volume and lower percentage of signal recovery than astrocytomas.*

*• Cerebral blood volume and percentage of signal recovery combined provide a broader perspective on tumor vasculature and yield promising results for this preoperative classification.*

**Supplementary Information:**

The online version contains supplementary material available at 10.1007/s00330-024-10611-z.

## Introduction

The imaging-based presurgical differentiation between astrocytomas and oligodendrogliomas represents an unsolved challenge in neuro-oncology. This differentiation has potential implications for diagnosis and initial patient management in several ways. First, accurate radiological classification allows for the selection of the most time- and cost-effective diagnostic test sequence in histopathology and molecular pathology [1–5]. In certain situations, it can further enhance the accuracy of the final diagnostic process. For instance, some researchers advocate for additional molecular testing for 1p19q co-deletion when there is a discrepancy between neuroimaging and fluorescence in situ hybridization results [5]. Secondly, the differentiation between these two entities assists in choosing the most appropriate surgical approach (e.g., total resection vs. partial resection or biopsies), predicting prognosis, and deciding the necessity and intensity of different adjuvant therapies, all of which can vary between astrocytomas and oligodendrogliomas [1–4].

Despite significant advances in imaging techniques, achieving accurate differentiation preoperatively remains complex. This emphasizes the need for innovative approaches that can enhance current diagnostic capabilities [6]. Qualitative morphological imaging, particularly through the T2-FLAIR mismatch sign, provides valuable insights and is highly specific for identifying IDH-mutant astrocytomas [7]. However, this method has low sensitivity, and its interpretation remains largely visual and subjective, reliant on the experience of the neuroradiologist with possible uncertain cases, especially for those less experienced. As a result, there has been an increasing interest in quantitative sequences. These offer the potential for more objective and reproducible results, enhancing the precision and reliability of presurgical tumor differentiation [8].

Dynamic susceptibility contrast perfusion-weighted imaging (DSC-PWI) is one such quantitative sequences that has received extensive attention in neuro-oncology. Widely available and included in the most recent brain tumor imaging consensus, DSC-PWI monitors T2* signal changes dynamically during the vascular passage of a gadolinium-based contrast agent (GBCA), thereby generating time-intensity curves. The standard evaluation involves calculating the area under the curves to obtain cerebral blood volume (CBV), which estimates the overall vascularization of the tumors [9–11]. However, the potential information embedded in these time-intensity curves goes beyond just CBV. For instance, the percentage of signal recovery (PSR) is another metric that has received significant attention and demonstrated promising results for differential diagnosis of brain tumors [12–14].

The PSR provides an indirect measure of T1 and T2* leakage effects, which notably influence the time-intensity curve after the first pass of GBCA. It has been extensively studied in tumors with well-known prominent blood-brain barrier (BBB) disruption like glioblastoma or lymphoma [13–20]. However, in the context of a more preserved BBB, such as in grade 2–3 gliomas [21], PSR biological meaning may be different and it is scarcely investigated.

Commonly, CBV (and PSR) calculations focus on mean values from two-dimensional (2D) regions of interest (ROIs), or whole-tumor three-dimensional (3D) volumes of interest (VOIs). Some authors suggest using extreme (maximum/minimum) values, either automatically generated or manually identified via hot-spots of CBV. Nevertheless, some of these methods have their shortcomings. First, 2D ROIs only evaluate isolated regions of the whole tumor and overlook tumor heterogeneity [12]. Second, relying on preselected single values, such as the mean or maximum, may overlook potential significant differences within the entire spectrum of voxel-wise values. These values can be further investigated using histogram-derived percentiles [22].

Finally, when CBV and PSR are evaluated, they are treated separately and their performances are compared as if one must choose between them. However, the reality is that they offer distinct information that characterizes tumor vascular-microvascular habitats from different perspectives. Therefore, CBV and PSR could be integrated, providing additive rather than mutually exclusive information [23].

Taking all these considerations together, the authors’ objectives in the context of pre-surgical DSC-PWI evaluation of astrocytomas and oligodendrogliomas are:

1. to characterize each tumor’s DSC-PWI features, assess the discriminative power of CBV and PSR, and compare the performance of common preselected values (mean, extreme) with unsupervised voxel-level percentile values derived from 3D tumors’ segmentations and
2. to unlock the potential value of combining CBV and PSR for these tumors’ pre-surgical differentiation.

## Material and methods

This retrospective study was approved by the Research Ethics Committee of Hospital Universitari de Bellvitge.

### Patients

Patients diagnosed with IDH-mutant astrocytomas and IDH-mutant 1p19q-codeleted oligodendrogliomas, grades 2 or 3, were retrospectively retrieved from our center’s database spanning the years 2010–2022. The stud’s inclusion criteria were as follows: (1) confirmed tumor diagnosis in accordance with the World Health Organization classification of CNS Tumors 2021 [24], and (2) availability of a diagnostic pre-surgical MR examination including DSC-PWI, T1WI, T2WI, FLAIR, and contrast-enhanced T1WI (CE-T1WI). The study’s exclusion criterion was sequences of such low quality that prevented tumor segmentation or DSC-PWI data extraction.

All tumors were classified following the WHO 2021 classification criteria. The classification process encompassed histopathological examination, immunohistochemical analysis for IDH, p53, and ATRX, as well as fluorescence in situ hybridization (FISH) for detecting 1p19q codeletion. The missing tests were retrospectively conducted for tumors with incomplete records using archived samples from the pathology department’s biobank at our hospital as part of a national research project.

### Imaging

All the MR imaging examinations included in the study were acquired in the same tertiary hospital with 2 different scanners: Ingenia 3.0- or Ingenia 1.5-T (Philips Healthcare). All DSC-PWI sequences were gradient-echo, with the following technical parameters: time of echo, 40ms; time of repetition, 1500–1700 ms; flip angle, 75°; pixel size, 1.75 mm^2^; slice thickness, 5 mm; matrix size, 128 × 128; number of slices, 20–25; number and duration of dynamics, 60 and 1.5s. A single dose of 0.1 mmol/kg of intravenous GBCA (1 mmol/mL) was injected at a rate of 4–5 mL/s. No preload was administered. The baseline was in the order of 10–15 points. The quality of the sequences was assessed by two experienced neuroradiologist, i.e., A.P-E. and C.M., with more than one and two decades of experience in neuro-oncological radiology. Technical details of the morphological sequences (T1WI, T2WI, and FLAIR) are summarized in Supplemental Material 1.

### Post-processing and DSC-PWI data extraction

The volumetric segmentations of the entire tumors, considering axial T1WI, T2WI, FLAIR, and CE-T1WI, were carried out semi-automatically (histogram thresholding) and checked by two experienced neuroradiologists: A.P-E. and C.M. The segmentations encompassed the entire signal abnormality in T2-FLAIR. The additional sequences were used as support to identify macroscopically normal brain vessels, cysts, calcifications, hemorrhage, or necrosis, which were all excluded from segmentations. Simultaneously, ten ROIs of 5-mm diameter were selected in the contralateral normal appearing white matter for normalization. The ten ROIs were consistently and homogeneously placed across all patients through a consensus approach by two experienced neuroradiologists (A.P-E. and C.M.). The ROIs were positioned within the centrum semiovale [25], covering two contiguous slices, each 5 mm in thickness. All segmentations and ROIs were done using 3D Slicer (http://www.slicer.org), and co-registered with DSC-PWI using the BRAINSFit module. The extraction of DSC-PWI metrics was streamlined using an in-house pipeline. CBV maps were produced with the DSC MRI analysis module in 3D Slicer. This software calculates CBV in accordance with consensus methodologies [11]. Time-intensity curves were converted into time-concentration curves and the area under the curve was calculated to obtain relative CBV, applying leakage correction using the Boxerman-Schmainda-Weiskoff (BSW) method. The PSR was captured early in the theoretical end of the first vascular pass of gadolinium on the time-intensity curve, automatically detected when the mean ascending slope of the curve fell below its standard deviation. This early PSR was then computed as described by Cha et al [26]. For each voxel within the tumors, normalized relative CBV leakage-corrected (from now on, nrCBV) and PSR were calculated. For each tumor, the mean, maximum, and minimum values of all the voxels as well as percentile values in increments of five were calculated.

### Description and comparison of DSC-PWI metrics

Statistical analyses were conducted to compare astrocytomas and oligodendrogliomas nrCBV and PSR mean, minimum, maximum, and percentiles via Mann-Whitney *U* test. Simultaneously, we calculated the area under the receiver operating characteristic curve (AUC-ROC) for all variables, with tenfold stratified cross-validation to enhance robustness [27]. Finally, box-plots were constructed to visually assess the segregation potential of all these variables. In addition, a subanalysis was performed to compare differences between histological grades within each tumor type statistically.

### Combination of DSC-PWI metrics percentile values

In order to estimate the potential for classification by jointly considering nrCBV and PSR, we investigated a classifier. First, the correlation between nrCBV and PSR data was analyzed with a Spearman test. Then, the variables were narrowed down by selecting the nrCBV and PSR values with AUC-ROC > 0.8 [28] and *p*-value < 0.005 [29]. To further refine the number of variables included in the model and avoid overfitting, we limited to five features. The number five was chosen as the optimal according to our sample size [30]. Specifically, the final 5 variables were selected by applying recursive feature elimination choosing the variables that maximized accuracy in an iterative procedure until we were left with the top 5 most important features. Ultimately, we developed a straightforward gradient boosting classifier using a tenfold stratified cross-validation (find the details in Supplemental Materials 2 and 3). Gradient Boosting was chosen due to its ability to automatically capture complex interactions among features, robustness to outliers, and adaptability to avoid overfitting [31]. Lastly, we calculated its AUC-ROC to gauge its classification potential. All statistical computations were performed making combined use of Python version 3.10.5, R version 4.1.3, and IBM SPSS Statistics version 25.

## Results

### Patients

**Fig. 1:**
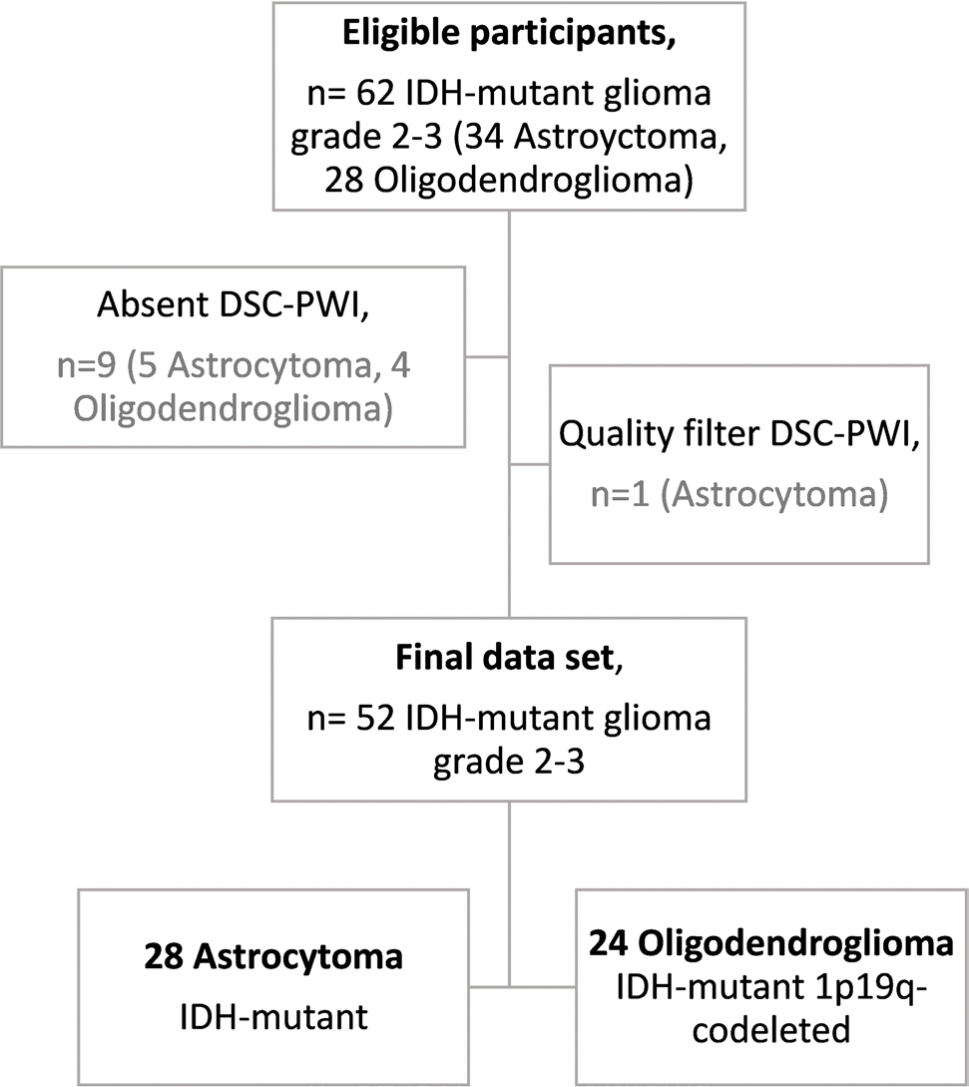
A flowchart summarizing the study participant selection process

Sixty-two candidates were initially identified for this study. Nine patients were excluded due to lack of DSC-PWI, and one was because of poor quality of DSC-PWI. The final participant count was 52, consisting of 28 astrocytomas and 24 oligodendrogliomas. Figure 1 provides a summary of the participant flowchart. The descriptive details, including age, sex, and grade, are compiled in Table 1, including statistical comparisons. The mean age for the entire dataset was 45 ± 13 years. Twenty-eight participants were men and 31 had grade 3 tumors. There were no significant differences between astrocytomas and oligodendrogliomas for the demographic variables.

**Table 1:** This table presents the demographic and clinical characteristics of the participants, including age, sex, and tumor grade. Statistical comparisons were made using the Mann-Whitney *U* test for age, and the chi-square test for both sex and tumor grade. *SD* standard deviation

	Oligodendroglioma	Astrocytoma	Whole dataset	*p* value
Age in years (mean ± SD)	49 ± 14	43 ± 13	45 ± 13	0.17
Sex, men: women	15:09	13:15	28:24	0.38
Grade, 2:3	10:14	11:17	21:31	0.85
Total	24	28	52	

### Description and comparison of DSC-PWI metrics

**Fig. 2:**
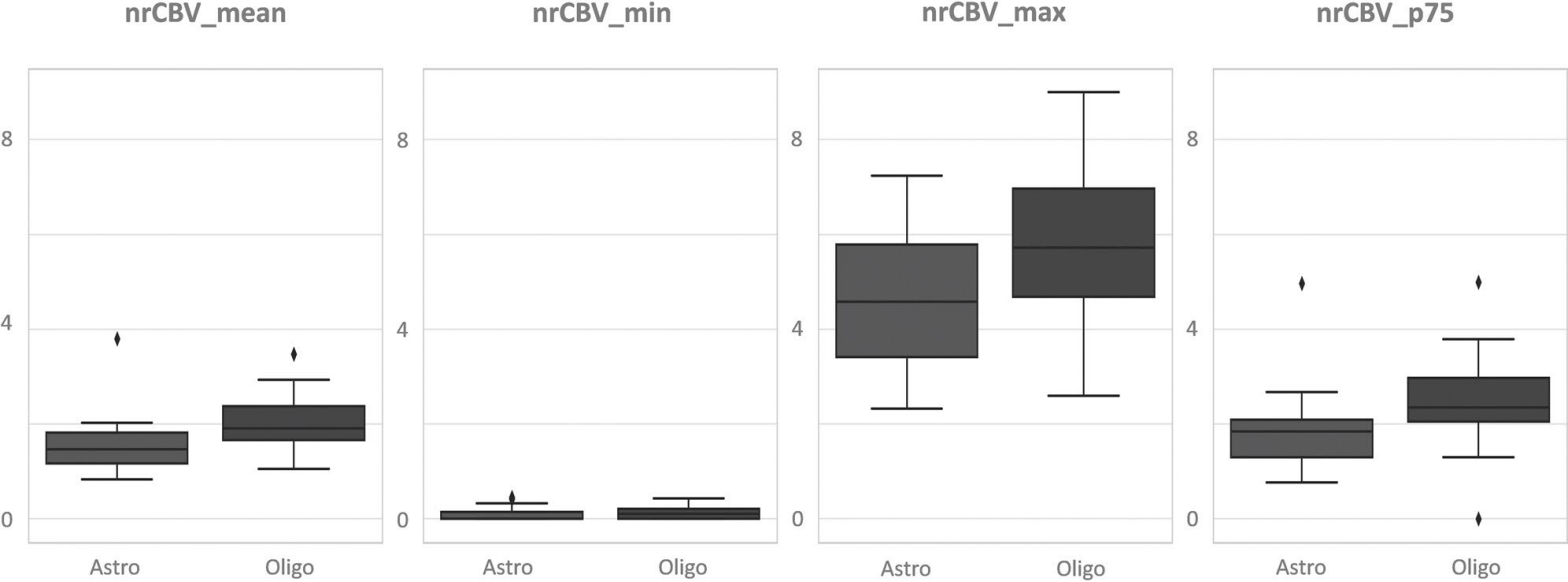
Whisker plots illustrate the distribution of all nrCBV values for each tumor subtype’s mean, minimum, maximum, and best percentile

**Fig. 3:**
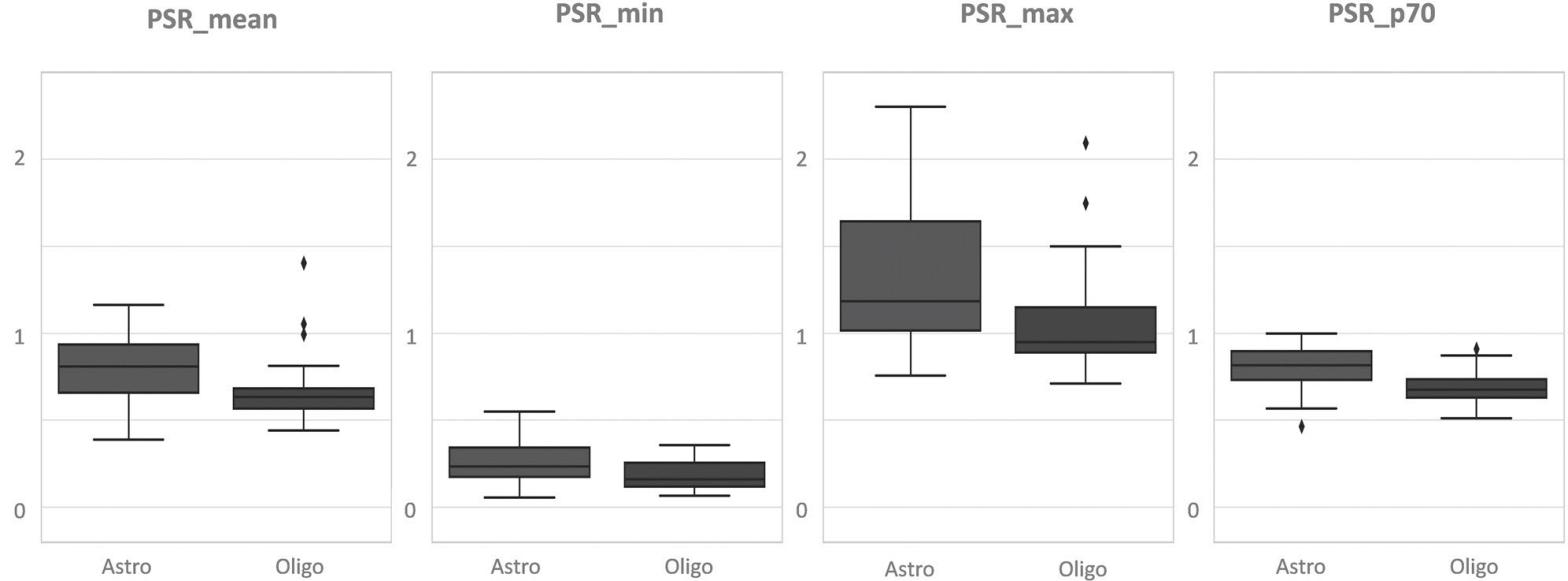
Whisker plots illustrate the distribution of all PSR values for each tumor subtype’s mean, minimum, maximum, and best percentile

Oligodendrogliomas exhibited higher nrCBV and lower PSR than astrocytomas across all metrics (e.g., mean nrCBV = 2.05 and 1.55, PSR = 0.68 and 0.81 respectively). The mean, minimum, maximum, and the best percentile values are shown in Table 2, along with their corresponding AUC-ROC and Mann-Whitney *U p* values. The highest AUC-ROCs derived from PSR percentile p70 (AUC-ROC = 0.84, *p* value = 0.0005) and nrCBV percentile p75 (AUC-ROC = 0.80, *p* value = 0.0006). The specificities for these two percentiles were 0.93 and 0.72, sensitivities of 0.65 and 0.80, and accuracies of 0.79 and 0.76, considering the optimal thresholds of 0.71 and 2.02, respectively. The AUC-ROCs for mean nrCBV (0.74) and PSR (0.73) were lower than that of several percentiles. The lowest results were observed for minimum and maximum values (AUC-ROCs from 0.64 to 0.69). A *Z*-test demonstrated statistically significant differences in AUC-ROCs of PSRp70 and nrCBVp75 above the minimum, maximum, and mean values (*p *< 0.035).The whole range of results, AUC-ROCs’ confidence intervals, and *p* values before and after the Bonferroni correction are available in Supplemental Material 4. It is also noteworthy that the best percentile values exhibited lower standard deviations and narrower confidence intervals, which further suggests an increased level of robustness in comparison to mean, minimum, and maximum. The distributions of all the values for each metric and group are depicted in box-plots in Figs. 2 and 3. These graphics clearly visualize the varying segregation potential, accentuated for percentile values.

**Table 2:** Summary of mean ± standard deviation values for nrCBV and PSR variables for each tumor subtype. These values correspond to the most common preselected mean, minimum, and maximum variables, and the best discriminating percentiles. The corresponding AUC-ROC and Mann-Whitney *U p* values are also included. * indicates statistical significance according to classic criteria (*p *< 0.05), and ** according to recently suggested more restrictive and robust criteria (*p *< 0.005)

**Mean**	**nrCBV**	**PSR**	**Minimum**	**nrCBV**	**PSR**
**Oligo**	2.05 ± 0.58	0.68 ± 0.21	**Oligo**	0.13 ± 0.13	0.19 ± 0.09
**Astro**	1.55 ± 0.58	0.81 ± 0.19	**Astro**	0.09 ± 0.14	0.27 ± 0.12
**AUC-ROC**	0.74	0.73	**AUC-ROC**	0.67	0.67
**Mann-*U*, *p***	0.001**	0.005*	**Mann-*U*, *p***	0.13	0.01*
**Maximum**	**nrCBV**	**PSR**	**Percentiles**	**nrCBV p75**	**PSR** **p70**
**Oligo**	5.91 ± 1.82	1.17 ± 0.60	**Oligo**	2.53 ± 0.98	0.69 ± 0.11
**Astro**	4.72 ± 1.75	1.33 ± 0.43	**Astro**	1.86 ± 0.78	0.81 ± 0.12
**AUC-ROC**	0.69	0.64	**AUC-ROC**	0.80	0.84
**Mann-*U*, *p***	0.01*	0.01*	**Mann-*U*,* p***	0.0006**	0.0005**

Of note, no significant differences in the Mann-Whitney *U* test were found for the nrCBV nor PSR values between grades 2 and 3 (Supplemental Material 5). Ultimately, 7 out of the 28 astrocytomas and 5 out of the 24 oligodendrogliomas were assessed using a 3.0-T scanner. The chi-square test verified that there were no significant differences in the distribution of 3.0-T studies between the two groups (Supplemental Material 6). Furthermore, when comparing DSC-PWI metrics between 1.5 T and 3 T within each tumor type, we found no significant differences attributable to the field strength employed.

### Combination of DSC-PWI metrics percentile values

The Spearman correlation analysis revealed a general lack of significant correlation between the nrCBV and PSR values (see the details in Supplemental Material 7). The nrCBV or PSR percentiles with AUC-ROC > 0.8 [28] and *p* value < 0.005 [29] were nrCBV percentiles p70 and p75 and PSR from p40 to p75. AUC-ROC values ranged from 0.80 to 0.84 and *p* values from 0.0002 to 0.0007. Of note, all these variables remain significant after Bonferroni correction (Supplemental Material 4). Following the recursive feature elimination, the five definitive variables selected for classification, in order of feature importance (specified in brackets), were PSR_p65 (0.366), CBV_p70 (0.236), PSR_p60 (0.202), CBV_p75 (0.119), and PSR_p40 (0.078). The mean AUC-ROC for the gradient boosting classifier was 0.87, with a standard deviation of 0.16 and 95% confidence intervals ranging from 0.84 to 0.91 outperforming all the previous single-metric and single-value performances (*Z*-test, *p *< 0.05). Finally, the best cross-validation fold produced an AUC-ROC of 0.91.

As a final addition to the main analysis, the best differentiating metrics of the study were evaluated by categorizing astrocytomas with or without T2-FLAIR mismatch. This sign, evaluated visually by two experienced neuroradiologists, was only present in 10 out of the 28 astrocytomas (36%). In the T2-FLAIR mismatch absent group, the AUCs of CBVp75, PSRp70, and the classifier were 0.78, 0.82, and 0.85 retaining a good to excellent performance.

## Discussion

**Fig. 4:**
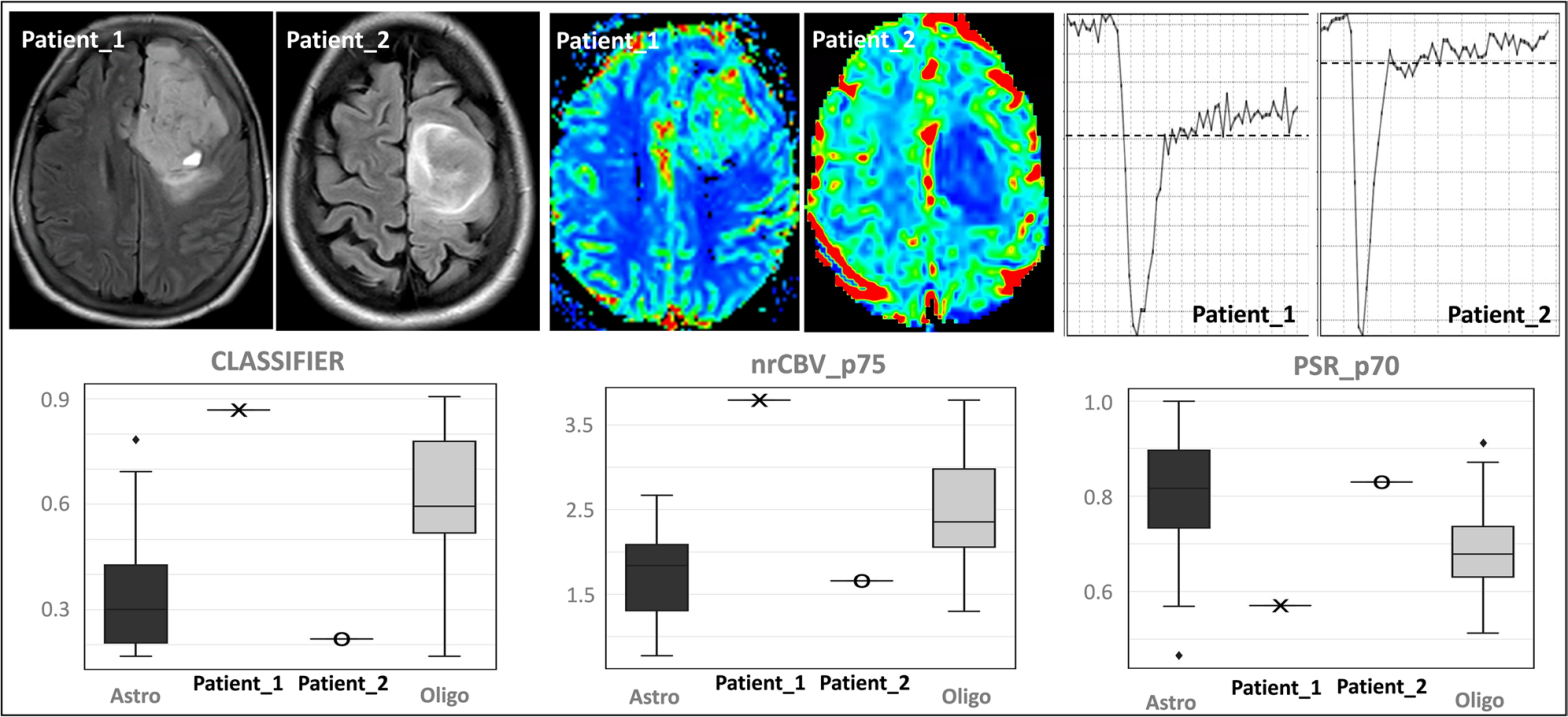
An illustrative example of the applicability of our proposed methodology for classifying new cases. Patient_1, a 64-year-old female, and Patient_2, a 34-year-old female, both studied using 1.5-T MR scan. Two FLAIR sequences of the patients show two frontal infiltrative tumors along with the rCBV maps and representative time-intensity curves. The classifier probability, nrCBVp75, and PSRp70 values of each case are overlaid onto the corresponding values of all astrocytomas and oligodendrogliomas in whisker plots. The display allows for a visual evaluation of the results, which suggests that Patient_1 would be more likely to be an oligodendroglioma and Patient_2 an astrocytoma. Both cases were histopathology confirmed. The histological grade was 3 in both cases

**Fig. 5:**
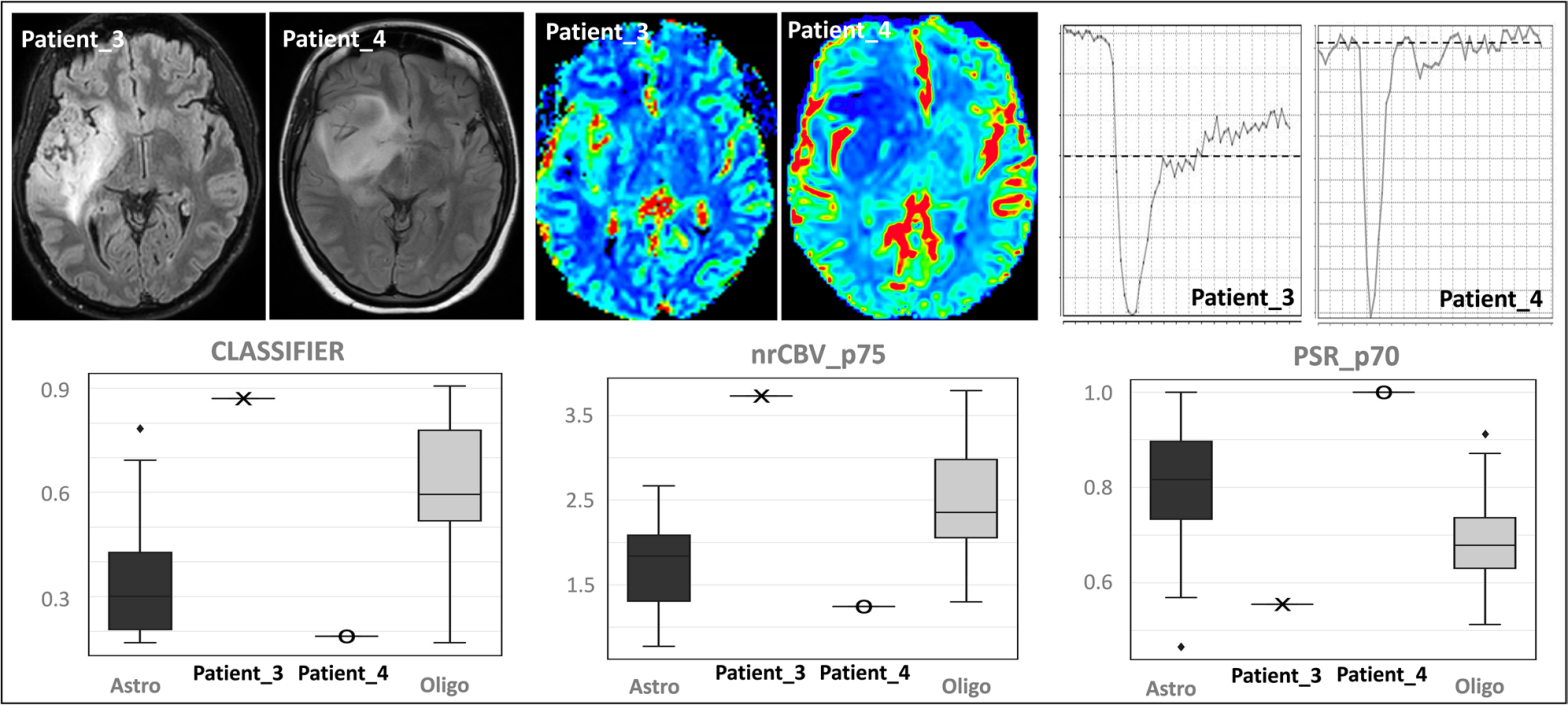
An illustrative example of the applicability of our proposed methodology for classifying new cases. Patient_3, a 42-year-old male, and Patient_4, a 25-year-old female, both studied using 3-T MR scan. Two FLAIR sequences of the patients show two fronto-insulo-temporal infiltrative tumors along with the rCBV maps and representative time-intensity curves. The classifier probability, nrCBVp75, and PSRp70 values of each case are overlaid onto the corresponding values of all astrocytomas and oligodendrogliomas in whisker plots. The display allows for a visual evaluation of the results, which suggests that Patient_3 would be more likely to be an oligodendroglioma and Patient_4 an astrocytoma. Both cases were histopathology confirmed. The histological grade was 2 in both cases

In this study, we delved into the distinctive features of DSC-PWI in distinguishing astrocytomas and oligodendrogliomas. Firstly, we have pioneered the exploration of the PSR as a potential discriminator between these tumor types. Our data indicates a lower PSR in oligodendrogliomas. Regarding nrCBV, our findings align with previous studies that observed higher nrCBV in this tumor type [32–36]. Furthermore, we have augmented the efficacy of PSR and nrCBV analysis by introducing an unsupervised method to identify the most discriminative voxel-wise percentiles (AUC-ROC PSRp70 = 0.84 and nrCBVp75 = 0.80), which exceeds the conventional approach of solely relying on single preselected values such as the mean or maximum. Notably, the combined use of nrCBV and PSR does not result in exclusivity, but rather in an additive effect, thereby forming a powerful basis for robust classification. An illustrative example of the applicability of our methodology to classify future presurgical patients is presented in Figs. 4 and 5.

The most common approach for nrCBV calculations involves focusing on the mean values of 2D ROIs manually depicted, or whole-tumor 3D VOIs. However, if a specific ROI is manually selected, operator dependency resurfaces, complicating reproducibility, and only a portion of the whole tumor is evaluated. Also, if only preselected values of the entire 3D tumor are used, the heterogeneity of the tumors (especially of oligodendrogliomas in the current differential) is overlooked. Indeed, relying on preselected values often assumes that they will capture the most significant differences, an assumption that may lack a robust foundation [12]. Therefore, these methods fail to consider that differences may exist anywhere across the full spectrum of voxel-wise values [22]. In this work, we propose an objective, unsupervised, voxel-level percentile evaluation of 3D segmentations of the whole tumor. This offers more reproducible, robust, and improved information that better captures tumor heterogeneity while avoiding weak assumptions in the preselection of optimal values to differentiate between entities.

In terms of novel insights about the PSR, to our knowledge, only two prior studies involved PSR evaluations in tumors with oligodendrocytic component. However, these studies were conducted prior to the establishment of the current 2021 WHO classification criteria and treated oligodendrocytic tumors as mixed with other low- and high-grade glial tumors [37, 38]. Therefore, their results are not applicable to the current classification system and are not comparable with ours. PSR uniquely provides indirect measures of the interplay between T1 and T2* effects. In general terms, lower PSR values indicate predominant T2* effects, while higher values suggest predominant T1 effects. Based on our current understanding of leakage effects, it is known that PSR is shaped by a complex interplay of BBB disruption, variations in cell size and density, and the size and tortuosity of vessels. This theory mainly applies to brain tumors with extensive BBB disruptions. These disruptions enable a portion of the GBCA to leak through the vessels and interact with the extravascular extracellular space (EES) molecules and adjacent cellularity, while another portion remains within the vessels, reflecting vascular features [39–41]. However, when dealing with brain tumors with a more preserved BBB, such as the entities under investigation, the GBCA does not so significantly leak, thus minimizing the influence of BBB permeability and cellularity on the PSR. Consequently, it is plausible that the PSR in these tumors, such as grade 2–3 gliomas, primarily offers insight into the vascular architecture [42]. In simplified terms for clarity, in such cases, increased vessel disorganization and tortuosity could partially retain intravascular gadolinium. This incomplete wash-out could prevent the signal intensity curve from fully recovering, leading to a lower PSR. It is noteworthy that PSR calculation is not universally standardized. However, this specific vascular characteristic may be enhanced when the metric is calculated early towards the end of the first vascular pass of contrast, as done in this study. At this point, any potential GBCA leakage due to eventual foci of partial BBB disruptions is expected to be minimized.

This theory is further supported by previous observations in CNS tumors that lack a BBB and exhibit prominent tortuous vasculature, such as hypervascular metastases, meningiomas, or hemangioblastomas. These tumors are well-known for presenting with low PSR, attributable to their distinctive vascular features. Despite the different underlying explanation, these tumors share one commonality with the tumors currently under investigation: the absence, for one reason or another, of BBB leakage [22, 23, 26]. At this point, in the case of astrocytomas and oligodendrogliomas, it is crucial to point out that the histological vascular characteristics of oligodendrogliomas are marked by a network of irregular and tortuous vessels, frequently described as a chicken-wire capillary pattern [43–45]. This vascular pattern diverges significantly from that found in astrocytomas which while also altered generally retains a greater similarity to normal brain tissue [46]. This knowledge, especially about the unique microvascular architecture of oligodendrogliomas, aligns with our observations.

On the other hand, when nrCBV and PSR are evaluated, they are generally treated separately and their performances are compared as if one must choose between them. However, the reality is that they offer distinct information that characterizes tumor vascular features from different perspectives. In fact, and especially if correction methods are applied to nrCBV, PSR should not influence nrCBV values and vice versa, as also evidenced by our correlation analysis. Given these considerations, nrCBV and PSR could be combined, providing additive rather than mutually exclusive information [12, 23]. Additionally, by considering both metrics, we are considering the tumors’ vascular-microvascular habitats from a broader perspective than what single variables allow. Specifically, our combined nrCBV and PSR classifier demonstrates promising potential, with an estimated AUC-ROC of 0.87, an excellent result which still requires further validation. Beyond improved classification results, the simultaneous consideration of multiple DSC-PWI variables offers a more comprehensive perspective of the tumor’s vascular characteristics than traditional single metrics do. Therefore, this approach is likely to be more robust, mitigating single metric variabilities and providing a more holistic assessment of the vascular characteristics of the tumors.

Additionally, our T2-FLAIR mismatch subanalysis suggests that our method retains good to excellent performance in those tumors where the T2-FLAIR mismatch is absent or uncertain (such as in scenarios involving inexperienced radiologists). These preliminary results, out of the main scope of the present study, show promise and deserve dedicated further studies.

The generalizability of our results may be influenced by the technique employed and various post-processing steps, which can significantly impact CBV and PSR values. Current efforts towards standardization, as suggested by Boxerman et al [11] in 2020, recommend using either full-dose preloaded 60° flip-angle or 30° without preload sequences. However, considering the recent introduction of these recommendations and the advancements in genetic classification as per the WHO 2021 guidelines, there is currently scarce comprehensive data. This scarcity impedes the comparison of our results with others obtained using the agreed-upon consensus sequences for our specific purpose. Speculatively, using these preloaded or low FA techniques could result in higher CBV and lower PSR values, by minimizing the T1 leakage effects to optimize nrCBV measures [11], provided other post-processing steps remain consistent[12]. However, the expected differences in the studied tumors should be minor than those seen in tumors with extensive BBB disruption, and ultimately, we also applied post-processing leakage correction [47]. Additionally, non-preloaded intermediate-high flip-angle sequences (such as used in this study) have proven useful and may be the preferred choice for some authors and clinicians, specifically for pre-surgical diagnosis [13, 16, 17, 48]. Lastly, our methodology should be adaptable to different DSC-PWI pulse-sequence parameters by simply adjusting the thresholds [14]. Regardless, further multicentric validations are needed.

On another hand, our study design grouping grades 2 and 3 is a common approach in radiological papers (usually under the term “lower grade gliomas”) [36] and it is supported by the absence of any significant difference between grades in this work, which is also aligned with other radiological studies [49, 50]. Furthermore, several clinical papers also suggest that the precise genetic classification of tumors has reduced the impact of histological grading on biological behaviors [51–54]. All these facts ensure the study objectives and results are the most clinically relevant according to current neuro-oncological diagnostic trends. Also, this design was chosen to strictly adhere to the current WHO classification molecular criteria and, lastly, to avoid excessive dataset fragmentation. At any rate, separated data for each tumor grade is provided as supplemental material for increased clarity and transparency of results.

Some alternative investigations leveraged radiomic features derived from DSC-PWI, yielding promising results with innovative methodology [55]. In contrast, our study concentrates on the specific differentiation between astrocytoma and oligodendroglioma, and employs well-established and clinically accessible metrics, which facilitates ease of understanding and practical application while forges a direct connection with the foundational histological and biological processes, potentially opening new research avenues.

This study has some limitations that must be considered. First, it is a single-site, retrospective study. However, this ensures data homogeneity, which is important for pilot and exploratory studies such as ours. Also, other DSC-PWI metrics such as mean transit time and cerebral blood flow may have a role, yet their implementation requires arterial input function determination, leading to increased complexity in calculations. In contrast, our study boasts notable strengths that deserve mention. First, all tumors are classified according to the most recent 2021 WHO Classification criteria. Our findings offer valuable clinically relevant and applicable insights from various perspectives. For instance, our study reinforces the necessity of semiautomatic and unsupervised assessment of the entire tumor percentile values, instead of assuming that mean or maximum values are the best options, a common practice in clinical settings. Notably, this observation could serve as a suggestion for software vendors to consider including these options in their packages. Additionally, we provide new insights into oligodendrogliomas’ vascular and microvascular features using a widely available clinical sequence. Moreover, our findings may be applied to other clinical questions, potentially opening new research avenues. In this regard, we acknowledge emerging DSC sequences and metrics that may offer improved information regarding permeability or vascular architecture. Nevertheless, these sequences have not yet been widely adopted in clinical practice. Ultimately, the foundational knowledge uniquely presented in this work paves the ground for their further implementation.

In conclusion, oligodendrogliomas demonstrate a higher nrCBV and lower PSR than astrocytomas, characteristics that are accentuated when voxel-wise percentile values are considered. We hypothesize that the lower PSR might reflect higher microvascular tortuosity and aligns with the well-established histological substrate of oligodendrogliomas, known as the chicken-wire capillary pattern. Lastly, the combination of nrCBV and PSR percentiles augments the value of standard single-metric and single-value evaluations, yielding promising classification results.

### Supplementary Information

Below is the link to the electronic supplementary material.

Supplementary file1 (PDF 755 KB)

## Abbreviations

BBB: Blood-brain barrier
CBV: Cerebral blood volume
DSC-PWI: Dynamic susceptibility contrast perfusion-weighted imaging
EES: Extravascular extracellular space
GBCA: Gadolinium-based contrast agent
IDH: Isocitrate dehydrogenase
PSR: Percentage of signal recovery
WHO: World Health Organization

## Declarations

### Guarantor

The scientific guarantor of this publication is Albert Pons-Escoda.

### Statistics and biometry

Several authors have significant statistical expertise: Albert Pons-Escoda, Alonso Garcia-Ruiz, Pablo Naval-Baudin, and Ignacio Martinez-Zalacain.

### Study subjects or cohorts overlap

Some study subjects have been previously reported in Proton MR spectroscopy shows improved performance to segregate high-grade astrocytoma subgroups when defined with the new 2021 World Health Organization classification of central nervous system tumors, European Radiology 2023, in-press .

### Methodology

• retrospective

• diagnostic study

• performed at one institution
