# Supplementary material for: Differentiating IDH-mutant astrocytomas and 1p19q-codeleted oligodendrogliomas using DSC-PWI: high performance through cerebral blood volume and percentage of signal recovery percentiles
Source: Eur Radiol. 2024 Jan 29;34(8):5320–30. doi: 10.1007/s00330-024-10611-z (PMC11255054; doi:10.1007/s00330-024-10611-z)

Differentiating IDH-mutant astrocytomas and 1p19q-codeleted oligodendrogliomas using DSC-PWI: high performance through cerebral blood volume and percentage of signal recovery percentiles

Supplemental material 1. Summary of technical details of the morphological sequences (T1WI, T2WI and FLAIR).

Sequence (3T)	Type (GRE/SE)	FA (º)	TR (ms)~	TE (ms)~	Matrix	Slice Thickness (mm)
**T1**	GRE	8	10	5	299x296	3
**T2**	SE	90	3000	95	480x400	3
**FLAIR**	SE	90	5500	300	220x217	3
**Sequence (1.5T)**	**Type (GRE/SE)**	**FA (º)**	**TR (ms)~**	**TE (ms)~**	**Matrix**	**Slice Thickness (mm)**
**T1**	SE	90	500	15	288x255	5
**T2**	SE	90	5000	100	288x238	5
**FLAIR**	SE	90	11000	140	288x160	5

Supplemental material 2. Python script for the recursive feature elimination.

```
import pandas as pd

from sklearn.ensemble import GradientBoostingClassifier

from sklearn.feature_selection import RFE


# Sample data loading (replace this with your actual data loading)

# Ensure to adjust the file path and sheet name according to your needs

df = pd.read_excel("path_to_your_file.xlsx", sheet_name="your_sheet_name")


# Specify the target variable

y = df['Astro/Oligo']
```

```python
# Specify the features X

# These specific features were chosen based on AUC-ROC > 0.8, p < 0.005

X = df[["cbv_p70", "cbv_p75", "psr_p40", "psr_p45", "psr_p50", "psr_p55", "psr_p60", "psr_p65",
"psr_p70", "psr_p75"]]


# Set up the Gradient Boosting model

gb = GradientBoostingClassifier(n_estimators=200, random_state=42)


# Apply RFE to select a specific number of features

selector = RFE(estimator=gb, n_features_to_select=5, step=1)

X_selected = selector.fit_transform(X, y)


# Show the selected features

selected_features = X.columns[selector.support_].tolist()

print("Selected features:", selected_features)
```

Supplemental material 3. Python script for the final classifier construction.

```python
import pandas as pd

from sklearn.ensemble import GradientBoostingClassifier

from sklearn.model_selection import StratifiedKFold, cross_val_score

from sklearn.pipeline import Pipeline

from sklearn.preprocessing import StandardScaler


# Load the dataset

file_path = 'path_to_your_file.xlsx'

df = pd.read_excel(file_path, sheet_name='Name')
```

```
# Define the target variable
y = df['Astro/Oligo']

# Define the features
# These specific features were chosen based on AUC-ROC > 0.8, p < 0.005,
# and a Recursive Feature Elimination (RFE) procedure, resulting in a maximum of 5 features
selected_features = ['cbv_p70', 'psr_p65', 'psr_p60', 'cbv_p75', 'psr_p40']
X = df[selected_features]

# Define the classifier
# The hyperparameters (learning_rate, max_depth, n_estimators) were tuned using GridSearchCV
gb = GradientBoostingClassifier(learning_rate=0.01, max_depth=1, n_estimators=200,
random_state=42)

# Define a pipeline for preprocessing and classification
pipeline = Pipeline([
    ('scl', StandardScaler()),  # Standardize features
    ('clf', gb),  # Classifier
])

# Define a stratified 10-fold cross-validation
cv = StratifiedKFold(n_splits=10, shuffle=True, random_state=42)

# Calculate cross-validated AUC-ROC and accuracy scores
roc_auc_scores = cross_val_score(pipeline, X, y, scoring='roc_auc', cv=cv, n_jobs=-1)
accuracy_scores = cross_val_score(pipeline, X, y, scoring='accuracy', cv=cv, n_jobs=-1)
```

```
# Print mean and standard deviation of scores

print(f"AUC ROC: {roc_auc_scores.mean():.3f} (+/- {roc_auc_scores.std():.3f})")

print(f"Accuracy: {accuracy_scores.mean():.3f} (+/- {accuracy_scores.std():.3f})")


# Print individual cross-validation scores

print("Individual AUC ROC scores: ", roc_auc_scores)

print("Individual Accuracy scores: ", accuracy_scores)


# Now, train the model on the whole dataset and get the feature importances

pipeline.fit(X, y)


# Get feature importances from the trained model

importances = pipeline.named_steps['clf'].feature_importances_


# Create a dataframe for visualization

importances_df = pd.DataFrame({

    'Feature': selected_features,

    'Importance': importances

})


# Sort the dataframe by importance

importances_df = importances_df.sort_values(by='Importance', ascending=False)


print("Feature Importances:")

print(importances_df)
```

Supplemental material 4. All range of values (mean for each variable) in astrocytomas and oligodendrogliomas, accompanied by AUC-ROCs and their 95% confidence intervals, and U-Mann Whitney p-values with and without Bonferroni correction. An asterisk (*) indicates AUC-ROC > 0.8, p < 0.005, and p-value after Bonferroni correction < 0.05.

		Astros	Oligos	AUC_ROC	CI	p-value	p-Bonferroni
nrCBV	Min	0.09	0.13	0.67	[0.63, 0.72]	0.1299	1
	p10	0.24	0.34	0.71	[0.65, 0.77]	0.0223	1
	p15	0.36	0.51	0.74	[0.7, 0.79]	0.011	0.7159
	p20	0.48	0.64	0.73	[0.68, 0.78]	0.0084	0.5485
	p25	0.58	0.77	0.71	[0.66, 0.76]	0.0051	0.333
	p30	0.67	0.89	0.73	[0.68, 0.78]	0.0024	0.1549
	p35	0.76	1.02	0.73	[0.68, 0.78]	0.0021	0.137
	p40	0.86	1.14	0.74	[0.69, 0.79]	0.0014	0.0942
	p45	0.95	1.28	0.77	[0.72, 0.81]	0.0011	0.0683
	p50	1.06	1.43	0.78	[0.74, 0.83]	0.0011	0.0729
	p55	1.17	1.59	0.78	[0.74, 0.83]	0.0011	0.0729
	p60	1.31	1.77	0.77	[0.72, 0.81]	0.0011	0.0683
	p65	1.45	1.98	0.77	[0.72, 0.81]	0.001	0.064
	p70	1.63	2.23	0.80*	[0.76, 0.84]	0.0007*	0.0430*
	p75	1.86	2.53	0.80*	[0.76, 0.84]	0.0006*	0.0376*
	p80	2.16	2.98	0.78	[0.74, 0.83]	0.0005	0.0351*
	p85	2.6	3.53	0.75	[0.7, 0.8]	0.0011	0.0683
	p90	3.27	4.37	0.73	[0.69, 0.78]	0.0018	0.1138
	Max	4.72	5.91	0.69	[0.65, 0.74]	0.0116	0.7544
	Mean	1.55	2.05	0.74	[0.7, 0.78]	0.0014	0.0942
PSR		**Astros**	**Oligos**	**AUC_ROC**	**CI**	**p-value**	**p-Bonferroni**
	Min	0.27	0.19	0.67	[0.59, 0.75]	0.0136	0.8811
	p10	0.37	0.27	0.69	[0.61, 0.76]	0.0099	0.6441
	p15	0.44	0.33	0.71	[0.63, 0.78]	0.0051	0.333
	p20	0.49	0.38	0.73	[0.66, 0.81]	0.002	0.1288
	p25	0.53	0.42	0.78	[0.72, 0.84]	0.0009	0.06
	p30	0.57	0.46	0.77	[0.71, 0.83]	0.0008	0.0492*
	p35	0.6	0.49	0.79	[0.74, 0.84]	0.0005	0.0306*
	p40	0.64	0.52	0.81*	[0.76, 0.85]	0.0004*	0.0286*
	p45	0.66	0.55	0.82*	[0.78, 0.87]	0.0003*	0.0175*
	p50	0.69	0.58	0.82*	[0.78, 0.87]	0.0002*	0.0151*
	p55	0.72	0.6	0.81*	[0.77, 0.86]	0.0003*	0.0201*
	p60	0.75	0.63	0.82*	[0.77, 0.86]	0.0003*	0.0201*
	p65	0.78	0.66	0.82*	[0.78, 0.86]	0.0004*	0.0232*

	**p70**	0.81	0.69	0.84*	[0.79, 0.88]	0.0005*	0.0306*
	**p75**	0.85	0.73	0.83*	[0.78, 0.87]	0.0006*	0.0402*
	**p80**	0.9	0.77	0.79	[0.75, 0.84]	0.0008	0.0525
	**p85**	0.96	0.83	0.78	[0.74, 0.83]	0.0016	0.1069
	**p90**	1.07	0.93	0.75	[0.71, 0.79]	0.0025	0.1646
	**Max**	1.35	1.17	0.64	[0.58, 0.7]	0.0122	0.7946
	**Mean**	0.81	0.68	0.73	[0.68, 0.78]	0.0051	0.333

Supplemental material 5. All range of values (mean for each variable) in astrocytomas and oligodendrogliomas categorized by grade 2 and grade 3, accompanied by U-Mann Whitney p-values.

**Astros**				**Oligos**			
	**Grade 2**	**Grade 3**	**p-value**		**Grade 2**	**Grade 3**	**p-value**
**CBVmin**	0.09	0.10	0.42	**CBVmin**	0.14	0.12	1.00
**CBVp10**	0.23	0.24	0.58	**CBVp10**	0.33	0.34	0.90
**CBVp15**	0.35	0.37	0.42	**CBVp15**	0.49	0.52	0.90
**CBVp20**	0.46	0.48	0.42	**CBVp20**	0.61	0.66	0.95
**CBVp25**	0.58	0.57	0.42	**CBVp25**	0.72	0.80	0.90
**CBVp30**	0.69	0.66	0.51	**CBVp30**	0.82	0.93	0.67
**CBVp35**	0.79	0.75	0.54	**CBVp35**	0.93	1.06	0.50
**CBVp40**	0.90	0.84	0.68	**CBVp40**	1.03	1.20	0.46
**CBVp45**	1.02	0.92	0.76	**CBVp45**	1.14	1.35	0.50
**CBVp50**	1.14	1.02	0.92	**CBVp50**	1.27	1.51	0.50
**CBVp55**	1.28	1.13	0.92	**CBVp55**	1.40	1.68	0.46
**CBVp60**	1.44	1.25	0.92	**CBVp60**	1.56	1.87	0.46
**CBVp65**	1.62	1.38	0.96	**CBVp65**	1.74	2.11	0.43
**CBVp70**	1.85	1.54	0.88	**CBVp70**	1.96	2.37	0.43
**CBVp75**	2.14	1.74	0.68	**CBVp75**	2.23	2.68	0.46
**CBVp80**	2.52	2.01	0.45	**CBVp80**	2.74	3.10	0.46
**CBVp85**	3.07	2.41	0.26	**CBVp85**	3.29	3.65	0.33
**CBVp90**	3.87	3.03	0.24	**CBVp90**	4.15	4.47	0.71
**CBVmax**	5.51	4.40	0.24	**CBVmax**	5.75	5.99	0.90
**CBVmean**	1.75	1.48	0.72	**CBVmean**	1.94	2.10	0.50
**PSRmin**	0.24	0.28	0.48	**PSRmin**	0.24	0.16	0.08
**PSRp10**	0.34	0.38	0.54	**PSRp10**	0.33	0.24	0.09

**PSRp15**	0.41	0.45	0.54	**PSRp15**	0.40	0.30	0.08
**PSRp20**	0.46	0.50	0.48	**PSRp20**	0.44	0.35	0.06
**PSRp25**	0.50	0.55	0.48	**PSRp25**	0.49	0.39	0.06
**PSRp30**	0.54	0.58	0.51	**PSRp30**	0.52	0.42	0.08
**PSRp35**	0.57	0.62	0.65	**PSRp35**	0.55	0.46	0.09
**PSRp40**	0.61	0.65	0.61	**PSRp40**	0.58	0.49	0.09
**PSRp45**	0.64	0.68	0.54	**PSRp45**	0.60	0.52	0.11
**PSRp50**	0.67	0.70	0.58	**PSRp50**	0.63	0.55	0.09
**PSRp55**	0.70	0.73	0.72	**PSRp55**	0.65	0.58	0.13
**PSRp60**	0.73	0.76	0.76	**PSRp60**	0.68	0.61	0.18
**PSRp65**	0.76	0.79	0.76	**PSRp65**	0.70	0.64	0.18
**PSRp70**	0.79	0.82	0.76	**PSRp70**	0.73	0.68	0.18
**PSRp75**	0.83	0.86	0.76	**PSRp75**	0.76	0.72	0.33
**PSRp80**	0.88	0.91	0.61	**PSRp80**	0.79	0.76	0.39
**PSRp85**	0.94	0.97	0.80	**PSRp85**	0.84	0.83	0.50
**PSRp90**	1.03	1.09	0.61	**PSRp90**	0.91	0.94	0.58
**PSRmax**	1.27	1.38	0.54	**PSRmax**	1.06	1.23	0.95
**PSRmean**	0.76	0.83	0.72	**PSRmean**	0.67	0.69	0.95

Supplemental material 6. All range of values (mean for each variable) in astrocytomas and oligodendrogliomas based on the MR scanner field-strength used, accompanied by U-Mann Whitney p-values.

**Astros**				**Oligos**			
	**1.5T**	**3T**	**p-value**		**1.5T**	**3T**	**p-value**
**CBVmin**	0.06	0.20	0.09	**CBVmin**	0.12	0.18	0.26
**CBVp10**	0.20	0.33	0.09	**CBVp10**	0.31	0.46	0.12
**CBVp15**	0.34	0.45	0.30	**CBVp15**	0.47	0.65	0.16
**CBVp20**	0.45	0.54	0.38	**CBVp20**	0.60	0.80	0.21
**CBVp25**	0.56	0.63	0.43	**CBVp25**	0.72	0.95	0.16
**CBVp30**	0.65	0.71	0.50	**CBVp30**	0.84	1.07	0.18
**CBVp35**	0.75	0.79	0.60	**CBVp35**	0.97	1.20	0.24
**CBVp40**	0.85	0.87	0.72	**CBVp40**	1.10	1.33	0.33
**CBVp45**	0.95	0.95	0.72	**CBVp45**	1.23	1.46	0.41
**CBVp50**	1.06	1.04	0.88	**CBVp50**	1.38	1.60	0.49

**CBVp55**	1.19	1.14	1.00	**CBVp55**	1.54	1.76	0.63
**CBVp60**	1.32	1.25	1.00	**CBVp60**	1.72	1.94	0.68
**CBVp65**	1.48	1.37	1.00	**CBVp65**	1.94	2.16	0.73
**CBVp70**	1.67	1.51	0.88	**CBVp70**	2.19	2.39	0.73
**CBVp75**	1.91	1.69	0.80	**CBVp75**	2.50	2.67	0.95
**CBVp80**	2.24	1.91	0.60	**CBVp80**	2.96	3.04	1.00
**CBVp85**	2.72	2.23	0.35	**CBVp85**	3.54	3.52	0.95
**CBVp90**	3.45	2.73	0.14	**CBVp90**	4.37	4.33	0.95
**CBVmax**	5.01	3.84	0.11	**CBVmax**	5.91	5.89	0.89
**CBVmean**	1.61	1.40	0.40	**CBVmean**	2.02	2.15	0.63
**PSRmin**	0.27	0.27	0.96	**PSRmin**	0.19	0.17	0.73
**PSRp10**	0.37	0.35	0.64	**PSRp10**	0.28	0.25	0.68
**PSRp15**	0.45	0.41	0.57	**PSRp15**	0.34	0.31	0.73
**PSRp20**	0.50	0.45	0.43	**PSRp20**	0.38	0.36	0.84
**PSRp25**	0.55	0.49	0.32	**PSRp25**	0.43	0.40	0.78
**PSRp30**	0.59	0.52	0.35	**PSRp30**	0.46	0.44	0.84
**PSRp35**	0.62	0.55	0.30	**PSRp35**	0.49	0.47	0.78
**PSRp40**	0.66	0.57	0.27	**PSRp40**	0.52	0.50	0.78
**PSRp45**	0.69	0.60	0.32	**PSRp45**	0.55	0.53	0.73
**PSRp50**	0.72	0.62	0.27	**PSRp50**	0.58	0.55	0.68
**PSRp55**	0.75	0.65	0.21	**PSRp55**	0.61	0.58	0.73
**PSRp60**	0.78	0.68	0.16	**PSRp60**	0.64	0.60	0.68
**PSRp65**	0.81	0.70	0.13	**PSRp65**	0.67	0.63	0.62
**PSRp70**	0.84	0.73	0.18	**PSRp70**	0.70	0.66	0.63
**PSRp75**	0.88	0.76	0.16	**PSRp75**	0.74	0.69	0.53
**PSRp80**	0.93	0.80	0.18	**PSRp80**	0.79	0.72	0.45
**PSRp85**	1.00	0.85	0.16	**PSRp85**	0.85	0.76	0.30
**PSRp90**	1.12	0.92	0.08	**PSRp90**	0.96	0.81	0.18
**PSRmax**	1.43	1.09	0.08	**PSRmax**	1.23	0.92	0.37
**PSRmean**	0.86	0.67	0.17	**PSRmean**	0.71	0.60	0.41

Supplemental material 7. Heatmap showing the Spearman correlation coefficients between CBV and PSR values. The mean correlation remained a mere -0.22 (range, -0.52 to -0.0046). Furthermore, in the most discriminative variables included in our analysis (CBV percentiles p70 and

p75; PSR from p40 to p75), the range of Spearman correlation and p-values was -0.25 to -0.21 and 0.08 to 0.13 respectively, indicating a minimal and non-significant statistical correlation.

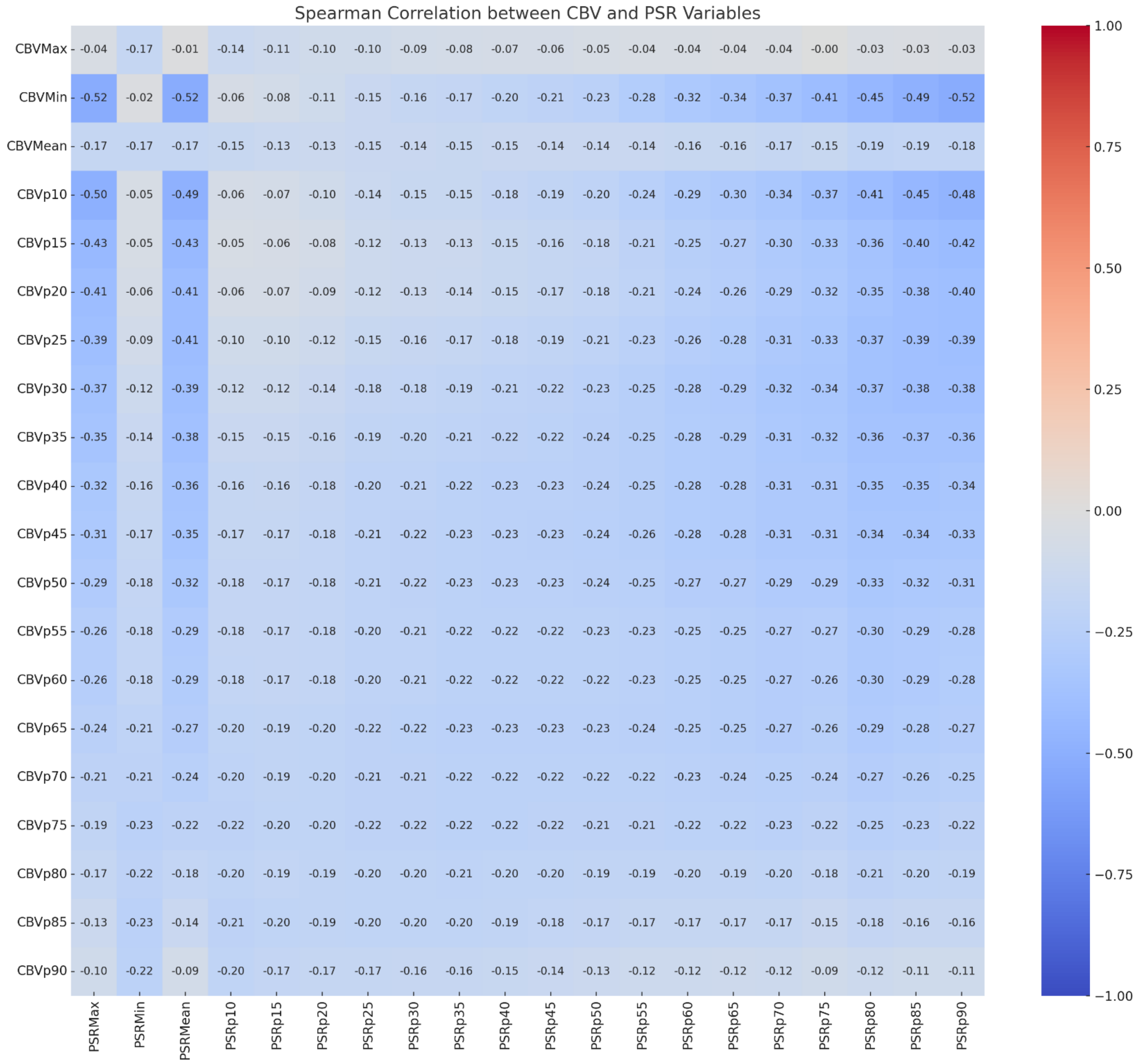

Supplement: Supplementary file 1 — Supplementary file1 (PDF 755 KB) [file 330_2024_10611_MOESM1_ESM.pdf]
